# Novel Artificial Tears Containing Cross-Linked Hyaluronic Acid: An In Vitro Re-Epithelialization Study

**DOI:** 10.3390/molecules22122104

**Published:** 2017-11-30

**Authors:** Arianna Fallacara, Silvia Vertuani, Giacomo Panozzo, Alessandra Pecorelli, Giuseppe Valacchi, Stefano Manfredini

**Affiliations:** 1 Department of Life Sciences and Biotechnology, Master Course in Cosmetic Science and Technology (COSMAST), University of Ferrara, Via L. Borsari 46, 44121 Ferrara, Italy; arianna.fallacara@unife.it (A.F.); mv9@unife.it (S.M.); 2 Ophthalmology Unit, Azienda ULSS n.22, 37012 Bussolengo, Italy; g.panozzo@iol.it; 3 Department of Animal Sciences, Plants for Human Health Institute, North Carolina State University, NC Research Campus, 600 Laureate Way, Kannapolis, NC 28081, USA; apecore@ncsu.edu (A.P.); valacc@ncsu.edu (G.V.)

**Keywords:** anti-inflammatory, artificial tears, corneal epithelium, cyclin D1, dry eye syndrome, HA, HA-CL, IL-8, re-epithelialization

## Abstract

Dry eye syndrome is a common disease which can damage the corneal epithelium. It is treated with eye drops to stimulate tear production and hydrate the corneal surface. The most prescribed artificial tear remedies contain hyaluronic acid (HA), which enhances epithelial wound healing, improving tissue health. To the best of our knowledge, only a few recent studies have investigated cross-linked HA (HA-CL) in eye drops for human applications. This work consists in an in vitro evaluation of the re-epithelialization ability of two different preparations containing a recently synthetized HA cross-linked with urea: 0.02% (*w*/*v*) HA-CL (solution 1, S1), and 0.4% (*w*/*v*) HA-CL (solution 2, S2). The study was conducted on both 2D human corneal cells (HCEpiC) and 3D reconstructed tissues of human corneal epithelium (HCE). Viability by 3(4,5-dimethylthiazol-2)2,5-diphenyltetrazolium bromide (MTT) test, pro-inflammatory cytokine release (interleukin-8, IL-8) by ELISA, and morphology by hematoxylin and eosin (HE) staining were evaluated. In addition, to understand the molecular basis of the re-epithelialization properties, cyclin D1 levels were assessed by western blot. The results showed no cellular toxicity, a slight decrease in IL-8 release, and restoration of epithelium integrity when the wounded 3D model was treated with S1 and S2. In parallel, cyclin D1 levels increased in cells treated with both S1 and S2.

## 1. Introduction

Dry eye syndrome or keratoconjunctivitis sicca (KCS) is a multifactorial disease of the tears and ocular surface, that results in symptoms of discomfort and visual disturbance, related to lacrimal film instability, with potential damage to the corneal epithelium [1]. It is accompanied by increased osmolarity of the tear film and inflammation of the ocular surface. When left untreated, this condition can provoke pain, ulcers, scars on the cornea, and even vision impairment [1]. Tear supplementation is the mainstay of the current therapy for the management of dry eye syndrome. This approach consists in the administration of artificial tears (which usually contain hydrophilic polymers) designed with a focus on physical properties relating to hydrating and lubricating of the ocular surface. Ideal tear replacement should recover and maintain a structurally and functionally normal ocular epithelium [1] and consequently improve patient ocular comfort and quality of life by alleviating and eliminating both the signs (objective) and symptoms (subjective) of the disease.

Natural tears have a particular rheological profile; they are viscous under static conditions in the eye, while they are much less viscous during blinking. This behaviour could be well reproduced by hyaluronic acid (sodium salt) eye drops. HA is a biocompatible and biodegradable polymer. It is a vital component of human ocular physiology: it naturally occurs in the vitreous, lacrimal gland, corneal epithelium and conjunctiva [2,3,4,5], and it has also been found in tear fluid [6,7,8]. HA has a unique viscoelastic profile. During blinks, shear stress causes HA molecules to align with each other. As a result, the solution momentarily loses its viscosity and spreads easily over the cornea surface. Between blinks, HA chains form a tangled meshwork, and the solution becomes more viscous. This stabilizes the pre-corneal tear film, and maximizes the solution residence time on ocular surface, where HA is able to improve eye hydration and lubrication, due to its hygroscopic and mucus-adhesive properties [9]. Moreover, HA has been shown to stimulate corneal epithelial cell migration, and to possess anti-inflammatory and antioxidant properties: consequently, it might play a role in wound healing [10,11,12,13,14]. The complete set of all these properties makes HA well suited for use in artificial tears. Many studies have been conducted to evaluate the safety and efficacy of HA solutions as eye drops. They have all highlighted appreciable improvements of KCS symptoms and signs, associated with HA concentration and molecular weight (generally 0.1–0.4% solutions of 0.8–1.4 MDa HA) [4,15,16,17,18,19,20]. All this explains why actually there are several HA-containing eye drops commercially available. However, most of the HA-containing artificial tears available on the market are characterized by the linear form of this polymer; there are only few and recent examples of eye drops consisting of HA-CL. Cross-linking is a chemical strategy with the aims to increase the rigidity of the polymer network (i.e., the gel viscoelasiticity), extend its permanence in the site of application and decrease its susceptibility to enzymatic degradation, thus reducing the daily number applications of a formulation [21]. To the best of our knowledge, only a few recent literature reports describe the effects of ophthalmic formulations –hydrogels, films, artificial tears-containing HA-CL for veterinary [22,23,24,25,26] and human uses [27,28,29,30]. These studies show promising results that open interesting perspectives to the ocular administration of cross-linked hyaluronans. Hence, the present work was devised with the aim to investigate the safety and the efficacy of eye drops based on a novel HA-CL to improve corneal re-epithelialization. More precisely, we examined the in vitro re-epithelialization capability of HA-CL preparations on both 2D and 3D human corneal epithelium model. In order to explore efficacy ranges, concentrations close to the ones of HA eye drops in the market, namely 0.02% and 0.4%, were assayed. The HA-CL used in this study is a recently patented polymer [31], provided with greater consistency as compared to naturally occurring hyaluronic acid [21,31]. This polymer consists of HA chains cross-linked by urea acting as a multifunctional agent [21,31]. Indeed, urea is not only a cross-linking agent –which increases native HA viscosity by linking its chains, thus possibly determining a longer retention on the corneal epithelium. Urea is also a non-toxic molecule with intrinsic healthy activity [21,31]. Therefore, urea-cross-linked hyaluronic acid is a promising polymer, because it has been developed not only to improve the mechanical properties of native HA, but also its biological activities [21,31]. In fact, urea is well known to be a moisturizing agent, thanks to its ability of water retention, which promotes cellular regeneration and reparation [21]. Charlton et al. [32] found that topical urea is able to encourage corneal re-epithelialization and to limit epithelial damage after injury to corneal epithelium. Therefore, all the beforehand mentioned studies about the ophthalmic use of HA and urea suggest that HA cross-linked with urea [31] could be an innovative promising ingredient for eye drops to induce corneal re-epithelialization. The herein described application of urea-crosslinked hyaluronan in the formulation of ophthalmic medicaments and medical devices is so far unprecedented. 

## 2. Results and Discussion

### 2.1. Synthesis of HA-CL 

**Scheme 1:**
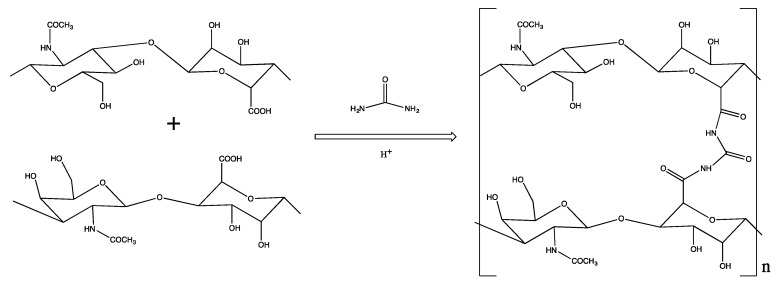
Synthesis of HA-CL with urea.

Hyaluronic acid cross-linked with urea (Scheme 1) was prepared as reported by Citernesi et al. [31].

### 2.2. Physical-Chemical Characterization and Stability of HA-CL Solutions

In this study, two prototypes of eye drops were obtained by gently dissolving HA-CL into a saline-buffered aqueous solution. Immediately after their preparation, both the solutions displayed a transparent and homogenous appearance, and pH and viscosity (η) values were respectively 7.0 ± 0.0 and 1.6 ± 0.0 mPa·s for S1, and 7.1 ± 0.0 and 85.9 ± 0.0 mPa·s for S2; therefore suitable for ophthalmic formulations.

The stability study showed that the both S1 and S2 met the intended physical and chemical quality standards, as well as functionality and aesthetics, when stored under appropriate conditions. Indeed, both the formulations appeared homogeneous and perfectly transparent during the whole test, maintaining their initial appearance under all the conditions. The pH and viscosity values were pretty much constant during the whole study (Table 1). Conservation at high temperature condition (40 ± 2 °C) provoked only extremely limited modifications of pH and viscosity parameters, which were more than acceptable (Table 1).

### 2.3. Cell Viability

Tear supplementation is the treatment of choice to control signs and symptoms of dry eye syndrome [1], and HA artificial tears eye drops are and among the most studied [4,10,11,12,15,16,17,18,19,20,29,30]. Corneal re-epithelialization, recovery and maintenance of ocular epithelium physiological conditions can be promoted also by urea therapy [32]. Hence, the novel hyaluronan derivative cross-linked with urea could be a valid therapy for KCS. Prior to specific markers screenings, the effect of HA-CL on human corneal cells viability was assayed. Table 2 reports cell viability results (MTT test) at 48 and 72 h, under the experimental conditions CTR−, CTR+ and treated with S1 and S2 solutions. As reported in Table 2, the wound caused a decrease of cell viability in damaged untreated tissues (CTR+); however, cell viability increased during time (Table 2). The damaged and treated tissues showed a trend similar to the positive control at 48 h, while, after 72 h of exposure, the cell viability increased to reach values comparable to negative control (not damaged) (CTR−) (Table 2).

Statistical analysis showed that after 72 h there were significant differences in cell viability between damaged untreated tissue (CTR+) and treated tissues (S1 and S2), and no significant differences between undamaged untreated tissue (CTR−) and treated tissues (S1 and S2) (Table 3). The viability parameter is correlated to cell proliferation that it is an index of the degree of tissue repair. Based on the results obtained, it was possible to conclude that both HA-CL solutions completely restored cell viability, thus promoting the re-epithelialization of the studied cellular model after 72 h.

### 2.4. Effect of HA-CL on IL-8 Levels

To understand if HA-CL solutions were able to modulate inflammation due to wound induction, IL-8 levels were quantified in human corneal control and treated cells. The chemokine IL-8 was chosen as pro-inflammatory marker as it is released in epithelial tissues in a pro-inflammatory status. Table 4 reports the results of IL-8 levels (ELISA test) at 48 and 72 h, in the experimental conditions CTR−, CTR+ and treated with S1 and S2 solutions. The wound caused an increased release, over time, of IL-8 in wounded untreated tissues (CTR+) (Table 4). The treated tissues (conditions S1 and S2) showed a trend similar to the positive control (CTR+) throughout the exposure period, with an increase in IL-8 release compared to the negative control tissues (CTR−) (Table 4).

Statistical analysis showed that there was no significant difference in IL-8 levels between the positive control (CTR+) and the treated tissues (S1 and S2), and also between the two treated tissues at both 48 h (Table 5) and 72 h post-treatment (data not shown).

Although no sample was able to significantly modulate IL-8 release in this particular experimental system, it was anyway possible to observe a slight reduction of IL-8 level in treated tissues compared to the positive control, especially at 48 h (−8.2% S1 vs. CTR+, −12.8% S2 vs. CTR+, Table 4) suggesting a possible positive effect of S1 and S2.

### 2.5. Epithelial Corneal Wound Closure

**Figure 1:**
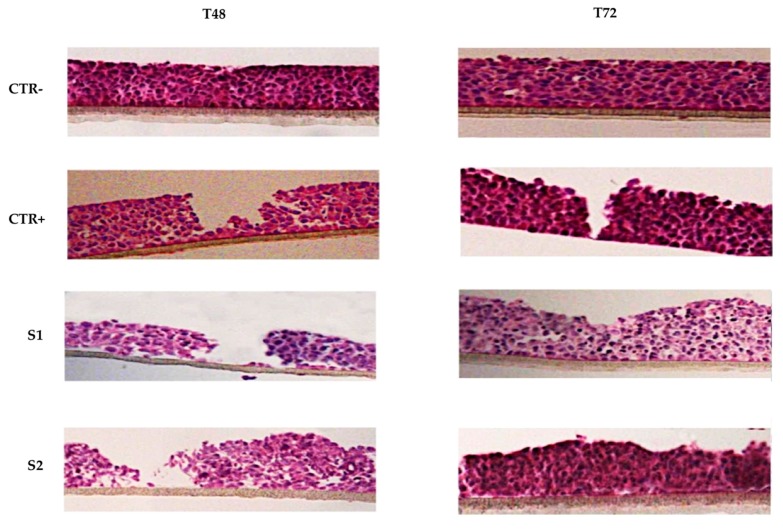
Histological analysis (HE staining) of 3D reconstructed tissues of human corneal epithelium. After wounding, HCE cells were incubated in growth medium with or without S1 and S2 for 72 h. Representative images from each group were recorded at 48 and 72 h post-wounding.

Histological analysis (HE staining) is a useful tool to study epithelial integrity and repair phenomena that follow the induction of mechanical damage. The histological images shown in Figure 1 present the overtime wound closure of the different samples. The negative control CTR− had an intact and correct structure for the entire period of exposure. After wound, it is possible to appreciate a recovery of the damage overtime in the positive control CTR+ samples at 48 h and 72 h. The epitheliums treated with the two solutions S1 and S2 exhibited a clear improvement in the wound closure respect to the positive control.

**Figure 2:**
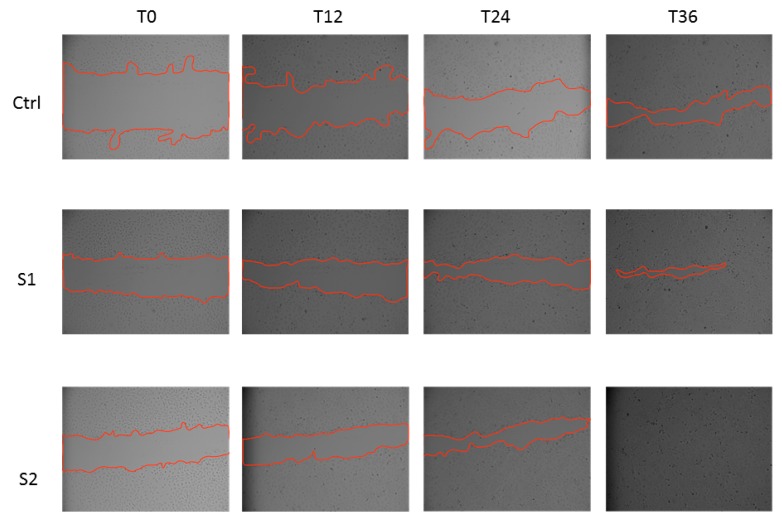
In vitro wound healing assay of human corneal epithelial cells. After the scratch, HCEpiC cells were incubated in fresh medium with or without S1 and S2 for 36 h. Representative images from each group were recorded at 0, 12, 24 and 36 h post-scratching. The red lines indicate the wound borders.

**Figure 3:**
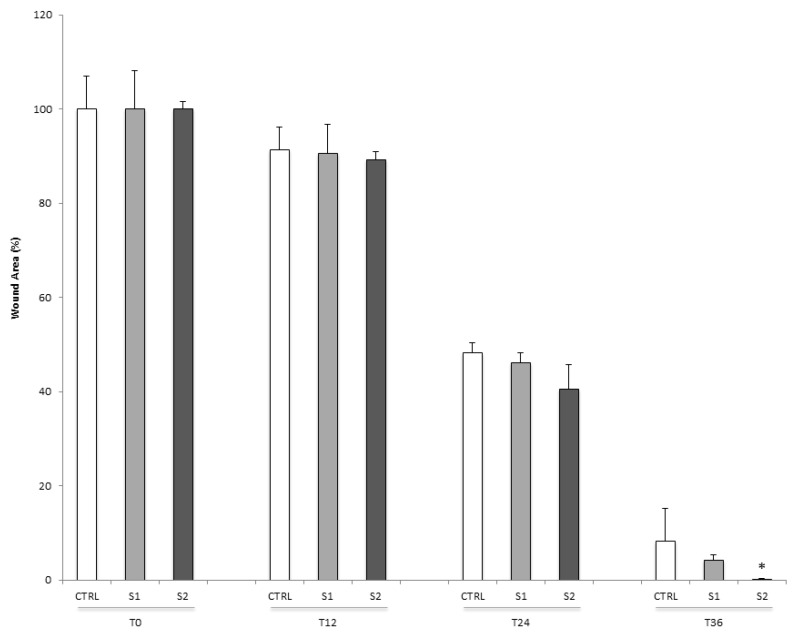
Quantification of wound healing in human corneal epithelial cells. The relative scratch gap was calculated as the percentage of the remaining wounded area at the given time point compared with the initially wounded area at 0 h. Data were expressed as mean ± SD (*n* = 3). * *p* < 0.05 compared with control.

To confirm the wound healing properties of S1 and S2, we also tested the two HA-CL solutions in a scratch assay on a 2D monolayer model of HCEpiC cells. Consistent with the 3D results, at 36 h post-scratch, treated HCEpiC cells displayed a nearly (S1) or complete (S2) wound closure compared to untreated control cells (Figure 2). In particular, a significant difference in wound closure was observed between S2 treated HCEpiC and untreated control cells (Figure 3), showing that S2 importantly improved epithelial corneal wound closure.

### 2.6. Cyclin D1 Protein Levels

**Figure 4:**
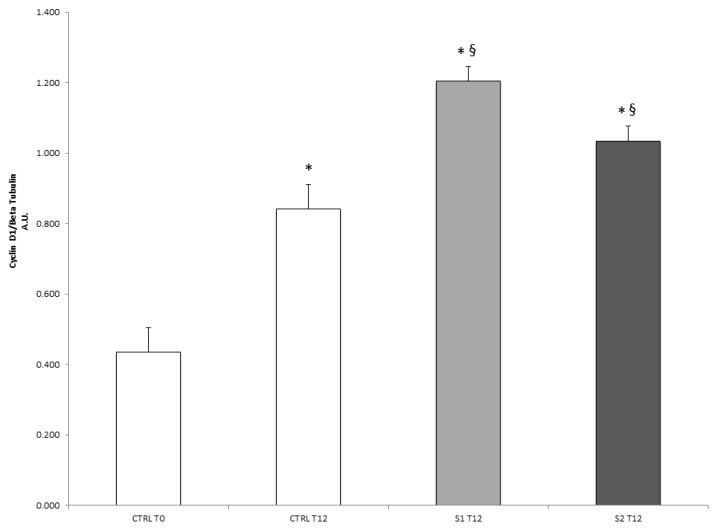
Effect of S1 and S2 on the proliferative marker cyclin D1 in HCEpiC cells. The graph shows the cyclin D1 protein levels in HCEpiC cells treated with S1 or S2 for 12 h. Data are means ± SD of triplicate. * indicates statistically significant difference from untreated control at 0 h; ^§^ indicates statistically significant difference from untreated control at 12 h (one-way ANOVA, *p* < 0.05).

To understand the molecular basis of the re-epithelialization properties of S1 and S2 in wound closure, the protein expression of cyclin D1, a cell-cycle regulator critical for G(1)-phase progression and S-phase entry, was also analyzed. As shown in Figure 4, both S1 and S2 treatments were able to induce a significant increase in cyclin D1 cellular levels after 12 h respect to the untreated control cells. This evidence suggested that S1 and S2 solutions might be able to accelerate epithelial wound closure by promoting a cyclin D1-induced cell proliferation.

## 3. Experimental Section

### 3.1. Materials

HA-CL with urea (*M*_w_ 2.0–4.0 MDa) was a patented raw material [31] kindly provided by IRAlab (Usmate Velate, Monza-Brianza, Italy). The solvent used for the preparation of HA-CL solutions was a saline-buffered solution consisting of Milli-Q water, NaCl, Na_2_HPO_4_∙12H_2_O, and NaH_2_PO_4_∙H_2_O (pH 7.0). All the salts were in compliance with European or USP Pharmacopoeia.

### 3.2. Formulation of HA-CL Solutions

Two different prototypes of solutions were formulated: 0.02% (*w*/*v*) HA-CL (solution 1, S1) and 0.4% (*w*/*v*) HA-CL (solution 2, S2). Each formulation was prepared by dissolving the polymer in the above described saline-buffered solution. The polymer was left to hydrate under gentle magnetic stirring, at room temperature, for about 1 h, until reaching a transparent and homogeneous appearance. The solutions were sterilized through 0.2 μm Stericup^®^ vacuum driven sterile filters (Millipore, Canton-Schaffhausen, Switzerland), and then they were stored at room temperature (23 ± 2 °C) before being evaluated.

### 3.3. Physical-Chemical Characterization and Stability of HA-CL Solutions

Immediately after their preparation, the two prototypes of solutions were characterized by measuring in triplicate their pH and their viscosity (η, rotational viscometer VISCO-STAR equipped with TL5 spindle, Fungilab, Barcelona, Spain) at room temperature (23 ± 2 °C).

Moreover, pH, η and macroscopic appearance of HA-CL solutions were monitored during time to evaluate the physical-chemical stability of the eye drops prototypes. After the preparation, each formulation was divided into two aliquots, one stored for six months at ambient temperature (23 ± 2 °C, shelf life), and the other stored for six months in thermostatic oven (40 ± 2 °C, accelerate stability test). At selected time intervals (i.e., 1 week, 1,2,3,6 months after the preparation), the samples were evaluated in triplicate for physical-chemical and organoleptic characteristics. Data were reported as mean values ± standard deviations.

### 3.4. In Vitro Efficacy Study

#### 3.4.1. Experimental Scheme

The adopted experimental scheme was:

- negative control condition: tissues not wounded and not treated (CTR−);
- positive control condition: tissues wounded but not treated (CTR+);
- treated condition: tissues wounded and treated with the samples (S1 and S2).

Four tissues for each experimental condition were used.

#### 3.4.2. Cell Cultures

The in vitro evaluations of the safety and the re-epithelialization capability of HA-CL eye drops prototypes were performed on two different biological models: 3D reconstructed tissues of HCE and 2D HCEpiC. 

The biological model consisting of 3D HCE, built from immortalized cells of human cornea (Model HCE–SkinEthic, Lyon, France) [33], was used for the viability study (MTT test), the determination of IL-8 release (ELISA test), and the morphological investigations (HE staining). The 3D HCE was a 0.5 cm^2^ corneal epithelium reconstructed by airlifted culture of transformed human corneal keratinocytes, placed for 5 days in chemically defined medium, on inert polycarbonate filter, at the air/liquid interface. After 6 days of reconstruction, corneal tissues were wounded by scalpel (with the exception of the negative control) and placed into plates with growth medium for the different treatments. 30 μL of test solutions (S1 and S2) were applied on the wounded tissues and incubated for 48 and 72 h at 36.5 °C/5% CO_2_. No substance was applied to negative and positive control tissues. Tissues were thus subjected to MTT test, ELISA test and HE staining.

The 2D biological model consisting of commercial HCEpiC cells was employed to confirm the wound healing properties of S1 and S2 (scratch assay), and to understand the molecular basis of the re-epithelialization in wound closure induced by S1 and S2 (cyclin D1 quantification by Western blot). HCEpiC cells grown in the corneal epithelial cell medium (ScienCell Research Laboratories, Inc., Carlsbad, CA, USA). Cells were incubated at 37 °C for 24 h in 95% air/5% CO_2_ until 80% confluence. The medium was changed every 4 days, and cells from passages 2–4 were used for experiments. Tissues were thus subjected to scratch assays and western blot tests. 

#### 3.4.3. Cell Viability

To evaluate the suitability, the safety and the effect on corneal cells viability of HA-CL eye drops prototypes, an in vitro MTT assay was conducted. After the treatment of 3D HCE cells as above described (Section 3.4.2), the tissues were rinsed three times with 1 mL of PBS, arranged in 300 μL of 0.5 mg/mL MTT solution and then incubated for 3 h at 36.5 °C/5% CO_2_. Each tissue was transferred into a well containing 1.5 mL of isopropanol and incubated for two h at room temperature. Hereafter, the tissues were removed from the wells and homogenized to dissolve formazan salts. Two hundred μL of this solution were transferred in a 96 well-plate and absorbance reading was performed at 570 nm (isopropanol was used as blank for reading). For each test condition the ratio of the average optical density of the treated tissues on the average optical density of negative controls determined the viability rate. Data were reported as mean values ± standard deviations (expressed in %), and as mean % variations compared to the controls.

#### 3.4.4. IL-8 ELISA Test

The inflammatory state of control and treated (S1 and S2) tissues was evaluated using ELISA test to quantify the pro-inflammatory marker interleukin-8 (IL-8). Therefore, after 3D HCE cells preparation as above described (Section 3.4.2), controls and treated tissues were undergone to IL-8 dosage using IL-8 ELISA commercial kit (ThermoFisher Scientific, Milano, Italy), according to the manufacturer′s instructions. For the quantitative determination, it was used a previously plotted calibration curve made-up of standard known and growing concentrations of IL-8. The results of IL-8 dosage in cell culture CTR−, CTR+ and treated with the two solutions at 48 and 72 h were reported as mean values ± standard deviations (expressed in pg/mL), and as mean % variations compared to the controls.

#### 3.4.5. Epithelial Corneal Wound Closure

Epithelial integrity and repair phenomena that followed wound induction were first of all studied on 3D HCE cells, and then confirmed on 2D HCEpiC. 

Control and treated tissues of 3D HCE were cultured and prepared as previously described (Section 3.4.2), and after stained with hematoxylin-eosin (HE) [34]. Microscope examination permitted to investigate the histology of the tissues. Indeed, hematoxylin, being a basic dye, coloured in blue/violet the negatively-charged cellular components principally located in the nucleus -nucleic acids, membrane proteins, cellular membranes, elastin-, while eosin, being an acid dye, stained in pink the positively charged cellular components predominantly situated in the cytoplasm and in the extracellular area -proteins, mitochondrial proteins, collagen fibers. The staining permitted to examine tissue integrity. 

To confirm the wound healing properties of S1 and S2, HCEpiC cells were subjected to wound healing assay performed as previously described [35]. Briefly, HCEpiC cells, grown to confluent monolayer on 48-well plates, were mechanically scratched with a 200-μL sterile pipette tip, washed and then allowed to re-epithelialize for 36 h in the presence of S1 and S2. Serial bright-field images of scratches were captured at different time points (i.e., 0, 12, 24 and 36 h post-scratch). Then, changes in wound area at various time points for each treatment group were measured using Image-J software (National Institutes of Health, Bethesda, MD, USA), and compared to the wound area at 0 h, which was arbitrarily set as 100.

#### 3.4.6. Western Blot Analysis

To evaluate cell proliferation, HCEpiC cells were seeded on cell culture dishes and, at subconfluence (50%), treated with the two solution S1 and S2. At 12 h, the cells were washed with ice-cold PBS and lysed in ice-cold lysis RIPA buffer. After centrifugation (15,000× *g*, 15 min at 4 °C), the supernatants were collected. Protein concentrations were determined using the Bio-Rad protein assay kit (Bio-Rad Laboratories, Inc., Hercules, CA, USA). Total protein extracts (20 µg) were loaded onto 10% sodium dodecyl sulphate–polyacrylamide electrophoresis gels and separated by molecular size. Gels were electro-blotted onto nitrocellulose membranes and then blots were blocked for 1 h in Tris-buffered saline, pH 7.5, containing 0.5% Tween 20% and 3% milk. Membranes were incubated overnight at 4 °C with Cyclin D1 antibody (Cell Signaling Technology, Inc., Danvers, MA, USA). The membranes were then incubated with horseradish peroxidase-conjugated secondary antibody for 1 h, and the bound antibodies were detected by chemiluminescence (Bio-Rad Laboratories, Inc.). β-actin (Cell Signaling Technology, Inc.) was used as loading control. Images of the bands were recorded with a ChemiDoc imaging system (Bio-Rad Laboratories, Inc.) and the densitometry analysis was performed using Image-J software. Results are expressed in arbitrary units as relative to β-actin expression.

#### 3.4.7. Statistical Analysis

Obtained data in the different experimental groups were subjected to statistical analysis and compared according to one-way ANOVA and Tukey-Kramer test. The variations were considered significant for *p* < 0.05.

## 4. Conclusions

Although the preliminary nature of this in vitro study, we have clearly shown, for the first time, promising results for the use of artificial tears containing HA-CL with urea for the treatment of dry eye disease and corneal injuries in human eyes. The two prototypes of eye drops developed were characterized by a good chemical-physical stability, and resulted safe for ophthalmic application. Despite both S1 and S2 were not able to significantly reduce IL-8 levels, they showed interesting wound healing properties. Indeed, according to the applied experimental protocol and to the data obtained, both the formulations showed a potential re-epithelialization efficacy on the cellular models analyzed: a clear recovery of the wound was observed in the 2D model and also in the 3D model. This was confirmed also by histological analysis, which showed the restoring of microscopic epithelial structure after treatment with S1 and S2. Our findings were in agreement with the results of previous in vitro and in vitro studies, which showed that corneal epithelial wound healing is promoted by native HA [10,11,12,36,37,38] and others type of HA-CL [22,23,24,25,26,27,28,29,30]. Moreover, western blot analysis evidenced that, after the treatment with S1 and S2 eye drops, the level of the proliferative marker cyclin D1 was increased compared to the control. Therefore, the two HA-CL solutions accelerated the tissue proliferative process related to post-wound re-epithelialization. This study opens encouraging perspectives, since HA-CL with urea may promptly alleviate both signs and symptoms of dry eye syndrome, even if used at concentration lower (0.02% *w/v*) than the usually employed for native HA artificial tears (generally 0.1–0.4%) [4,15,16,17,18,19,20,36]. Therefore, HA-CL with urea eye drops could allow a rapid improvement of patient ocular comfort and quality of life through a therapy with absolutely acceptable costs. All these evidences and considerations strongly support further investigations, both in vitro and in vitro, for a deeper characterization of the biological activity of artificial tears containing HA-CL with urea. Moreover, further researches will be necessary to understand the ocular surface residence time and thus the required dose-frequency of eye drops containing HA-CL with urea, as well as their potential delivery through the membrane.

## Figures and Tables

**Table 1:** Stability of S1 and S2 during 6 months, at 23 ± 2 °C and at 40 ± 2 °C: pH and viscosity values.

Time	T (°C)	S1		S2	
		pH	η (mPa∙s)	pH	η (mPa∙s)
Day 7	23 ± 2	7.0 ± 0.0	1.6 ± 0.0	7.1 ± 0.0	85.9 ± 0.0
	40 ± 2	7.0 ± 0.0	1.6 ± 0.0	7.1 ± 0.0	85.8 ± 0.0
Month 1	23 ± 2	7.0 ± 0.1	1.6 ± 0.0	7.0 ± 0.0	85.9 ± 0.0
	40 ± 2	7.0 ± 0.0	1.6 ± 0.0	7.0 ± 0.0	85.8 ± 0.2
Month 2	23 ± 2	7.0 ± 0.0	1.6 ± 0.1	7.0 ± 0.1	85.9 ± 0.0
	40 ± 2	7.0 ± 0.0	1.6 ± 0.2	7.0 ± 0.2	85.8 ± 0.1
Month 3	23 ± 2	7.0 ± 0.0	1.6 ± 0.0	7.0 ± 0.0	85.9 ± 0.0
	40 ± 2	7.1 ± 0.1	1.5 ± 0.0	7.0 ± 0.0	85.6 ± 0.3
Month 6	23 ± 2	7.0 ± 0.1	1.6 ± 0.0	7.0 ± 0.0	86.0 ± 0.0
	40 ± 2	7.1 ± 0.0	1.5 ± 0.2	7.0 ± 0.0	85.7 ± 0.0

**Table 2:** Cell viability (MTT test) of CTR−, CTR+, S1 and S2 at 48 and 72 h.

Condition	Cell Viability		% Variation vs. CTR−		% Variation vs. CTR+	
	48 h	72 h	48 h	72 h	48 h	72 h
CTR−	100.0 ± 0.5	100.0 ± 8.4	-	-	-	-
CTR+	72.4 ± 2.2	81.8 ± 4.4	−27.6	−18.2	-	-
S1	76.4 ± 2.1	107.2 ± 0.5	−23.6	+7.2	+4.0	+25.4
S2	74.4 ± 0.7	102.3 ± 2.0	−25.6	+2.3	+1.9	+20.5

**Table 3:** ANOVA and Tukey-Kramer test statistical analysis of cell viability (MTT test) of the conditions CTR−, CTR+, S1 and S2 at 48 and 72 h (statistically significant values in bold, *p* < 0.05).

Condition	vs. CTR−		vs. CTR+		vs. S1		vs. S2	
	48 h	72 h	48 h	72 h	48 h	72 h	48 h	72 h
CTR−	-	-	**0.00000**	**0.00983**	**0.00000**	0.41310	**0.00000**	0.97145
CTR+	**0.00000**	**0.00983**	-	-	0.07789	**0.00116**	0.58301	**0.00474**
S1	**0.00000**	0.41310	0.07789	**0.00116**	-	-	0.52885	0.72684
S2	**0.00000**	0.97145	0.58301	**0.00474**	0.52885	0.72684	-	-

**Table 4:** IL-8 levels in CTR−, CTR+, S1 and S2 tissues assessed at 48 and 72 h by ELISA test.

Condition	IL-8 pg/mL		% Variation vs. CTR−		% Variation vs. CTR+	
	48 h	72 h	48 h	72 h	48 h	72 h
**CTR−**	616.5 ± 37.7	595.7 ± 35.9	-	-	-	-
**CTR+**	762.2 ± 40.3	720.7 ± 49.5	+23.6	+21.0	-	-
**S1**	699.5 ± 32.4	701.5 ± 36.4	+13.5	+13.8	−8.2	−2.7
**S2**	664.3 ± 52.3	714.5 ± 58.2	+7.8	+15.9	−12.8	−0.9

**Table 5:** ANOVA and Tukey-Kramer test statistical analysis of IL-8 levels in CTR−, CTR+, S1 and S2 tissues at 48 h (statistically significant values in bold, *p* < 0.05).

Condition	48 h			
	vs. CTR−	vs. CTR+	vs. S1	vs. S2
CTR−	-	**0.00625**	0.15548	0.61723
CTR+	**0.00625**	-	0.37554	0.07523
S1	0.15548	0.37554	-	0.82461
S2	0.61723	0.07523	0.82461	-

## Abbreviations

The following abbreviations are used in this manuscript:

CTR−	negative control condition
CTR+	positive control condition
ELISA	enzyme-linked immunosorbent assay
HA-CL	cross-linked hyaluronic acid
HA	hyaluronic acid
HCE	3D reconstructed tissues of human corneal epithelium
HCEpiC	2D human corneal epithelial cells
HE	hematoxylin and eosin
IL-8	interleukin-8
KCS	keratoconjunctivitis sicca
MTT	3(4,5-dimethylthiazol-2)2,5 difeniltetrazolium bromide
S1	solution 1, 0.02% (*w*/*v*) HA-CL
S2	solution 2, 0.4% (*w*/*v*) HA-CL
